# Impact of the COVID-19 Pandemic on Autistic Adults: a Scoping Review

**DOI:** 10.1007/s40474-023-00268-6

**Published:** 2023-01-31

**Authors:** Anke M. Scheeren, Laura Crane, Melanie Heyworth, Elizabeth Pellicano

**Affiliations:** 1 Vrije Universiteit Amsterdam, Van der Boechorststraat 7, 1081 BT Amsterdam, The Netherlands; 2 Centre for Research in Autism and Education (CRAE), University College London, London, UK; 3 Reframing Autism, Sydney, Australia; 4 Macquarie School of Education, Macquarie University, Sydney, Australia; 5 Department of Clinical, Educational and Health Psychology, University College London, London, UK

**Keywords:** Autism, Autistic adults, COVID-19, Lockdown, Mental health, Wellbeing

## Abstract

**Purpose of Review:**

The COVID-19 pandemic and its associated restrictions have had a significant impact on people’s everyday lives, including the lives of Autistic adults. We aimed to (a) synthesise all papers currently published on the impact of the COVID-19 pandemic on autistic adults and (b) identify lessons for the care and support of Autistic adults in pandemic and post-pandemic times.

**Recent Findings:**

Fifty-five papers met the inclusion criteria. Most studies focused on the pandemic’s impact on the wellbeing of Autistic adults. Several studies focused on the use of (telehealth) services or the risk of COVID-19 infection/hospitalisation.

**Summary:**

Autistic adults were significantly impacted by the pandemic, both directly as indicated by higher COVID-19 infection and hospitalisation rates, but also indirectly due to severe service disruptions and social restrictions. Even though there were large differences observed both between as well as within individuals in terms of the negative/positive effects of the COVID-19 pandemic, most studies reported a negative effect on Autistic adults’ mental health. We draw several lessons from this review for the future care and support of Autistic adults, all of which must be underpinned by participatory research methods, that is, where Autistic community members are actively involved in setting research questions, testing the acceptability of the methods and interpreting and disseminating the results.

**Supplementary Information:**

The online version contains supplementary material available at 10.1007/s40474-023-00268-6.

## Introduction

The catastrophic impacts — both direct and indirect — of the COVID-19 pandemic on the lives of millions of people across the world are becoming increasingly clear. Some of these impacts have been the direct consequence of the virus itself, including a significant rise in serious acute ill-health and early death, to which disabled people [1], including Autistic people,^1^ may have been particularly at risk [5, 6]. Other impacts have been caused by the measures put in place to try to curb the spread of the virus, including restricted access to face-to-face health and other social services; the closure of schools and other educational institutions; and the frequent requirement for people to stay at, and work from, home to dramatically constrain their physical and social interactions — measures that have varied substantially across and within regions [7]. Some of these measures may have had occasional positive effects above and beyond their impact on slowing the spread of the virus [8]. Yet, there are good reasons to believe that these measures imposed a particularly heavy burden on Autistic people [5, 6], especially as this group is at greater risk of experiencing mental health problems [9, 10] and may depend significantly on formal and informal support from others to maintain quality of life.

The current scoping review sought to synthesise evidence of the impact of the COVID-19 pandemic on the lives of Autistic adults. We focus on Autistic adults specifically given that they are an under-represented group in current autism research [11, 12]; are more likely to have co-occurring physical and mental health conditions [10, 13•], which place them at risk of health complications following COVID-19 infection; and have poorer life outcomes across many domains, often due to systemic and social failures to provide the requisite support [14].

## Method

We addressed the following research questions:

1. What has been the impact of the COVID-19 pandemic on Autistic adults?
2. Which individual and/or contextual factors during the COVID-19 pandemic are associated with a more positive outcome for Autistic adults?

Based on research on the impact of the pandemic, we sought to identify lessons for the future care and support of Autistic adults. The review protocol was registered on Open Science Framework (10.17605/OSF.IO/B73QN).

### Eligibility Criteria

We used the following criteria to determine study eligibility:

1. An empirical quantitative and/or qualitative study on the impact of the COVID-19 pandemic and its associated restrictions on the mental health or wellbeing of at least one Autistic adult (> 18 years), based on self-report and/or observation/proxy-report. Autistic adult(s) should be mentioned explicitly in the paper’s “Method” section.
2. The sample should consist of adult(s) with a clinical diagnosis of autism or who self-identify as Autistic, and/or consists of informants for the Autistic adults. In cases where the sample also included Autistic children (< 18 years) or people with diagnoses other than autism (e.g. intellectual disability), the study is included only where Autistic adults’ data (a) are described separately in the paper or (b) make up ≥ 50% of the total sample.
3. English language.
4. Published in a peer-reviewed journal or as a pre-print on a pre-print service provider.

### Search Strategy

On 24–25th May 2022, we searched through the electronic databases of PubMed, EMBASE, CYNAHL, Psychinfo and Science Citation Index (Web of Science) using the following search terms: [1] COVID-19 OR coronavirus OR SARS-CoV-2 OR pandemic AND [2] autis* OR developmental disorder AND [3] adult. Additionally, we searched through the major autism-related journals, including *Autism*, *Autism Research*, *Journal of Autism and Developmental Disorders*, *Molecular Autism*, *Autism in Adulthood*, *Research in Autism Spectrum Disorders* and *Research in Developmental Disabilities* using the following search terms: [1] COVID-19 OR coronavirus OR SARS-CoV-2 OR pandemic AND [2] adult. On 8th August 2022, we screened the same databases and journals for any newly published journal articles or pre-prints in the period from 26th May to 8th August.

### Data Extraction

Table 1 shows key information extracted from eligible papers (where available).

**Table 1:** Data extraction form for selected papers

Author (year)	Type of study	Context (country, timing and Containment and Health Index, CHI^a^)	Sample	Comparison data	Outcomes (measures)	Mental health change over time	Results/Predictors better outcome	Community involvement	Major theme
Adams et al. (2022)	Quantitative and qualitative; retrospective	Where: UK When: August 2020–April 2021 CHI: 65.48–63.45	Clinicians: n = 55; 66% 25–39 years; 73% female gender	Comparison group: no; pre-lockdown data: no	Telemental health concerns and barriers (non-standardized survey; clinician-report)	N/A	Usefulness telemental health increased in case of no co-occurring diagnoses (ADHD/ODD/learning/cognitive problems), younger age, technological affinity	Not reported	Impact on services
Adams et al. (2021)	Quantitative; prospective and retrospective	Where: US When: T0: March 11–20, 2020; T1: May 18–27, 2020 CHI: T0: 25.60–59.52 T1: 66.67–66.67	Autistic adults: *n* = 275; M age = 26.5 years; age range: 18–35 years; 49% female sex; 43% female gender; 48% not employed^b^	Comparison group: no; pre-lockdown data: yes	Prospective: depression, anxiety, stress (DASS-42; self-report); Retrospective: COVID-19-related distress (non-standardized survey; self- report)	Retrospective: yes, decrease; Prospective: no change	Retrospective: 66% endorsed some COVID-19-related distress; prospective: being male, lower anxiety at T0, lower COVID-19-related distress at T1 predicted better outcome	Yes: study approval	Impact on wellbeing
Ali et al. (2022)	Qualitative retrospective	Where: UK When: Early 2021 CHI: -	Autistic adults (self-report): *n* = 11; age range: 27–67 years; 55% female gender Autistic adults (proxy-report): *n* = 7; age range: 18–25 years; 43% female gender Service providers: *n* = 6; 67% female gender	Comparison group: no; pre-lockdown data: no	Telehealth experience (interview; self-report/proxy-report)	N/A	Telehealth has benefits (no travel costs) and disadvantages (lacking internet; uncertainty about privacy); telehealth experience depends on personal qualities of healthcare provider; not suitable in cases of high distress, physical examinations or ID; healthcare system deemed rigid; autistic people experienced even higher barriers to access healthcare during the pandemic	Yes: autistic researchers	Impact on services
Bal et al. (2021)	Quantitative; prospective	Where: US When: T0: March 30–April 19, 2020; T1: May 27–June 6, 2020 CHI: T0: 63.10–66.67 T1: 66.67–66.67	Autistic adults: *n* = 396; M age = 37.4 years; age range: 18–74 years; 61% female sex; 14% not employed; 26% living alone	Comparison group: no; pre-lockdown data: no	Impact COVID-19 (non-standardized survey; self-report); Psychological distress (non-standardized survey; self-report)	Impact: yes, more life areas affected; Psychological distress: no change	Being male, older, no prior mental health condition, no COVID-19 experience, more hopefulness predicted better outcome (i.e. less psychological distress and fewer life areas affected)	Yes: survey design	Impact on wellbeing
Bleszynski et al. (2022)	Qualitative retrospective	Where: Poland When: October 22–November 13, 2020 CHI: 40.77–69.05	Autistic adults: *n* = 10; age range: 25–45 years; 60% female gender; 20% not employed	Comparison group: no; pre-lockdown data: no	Impact COVID-19 on social interactions (interview; self-report)	Individual differences	Autistic adults reported a range of social needs; some missed social contacts, some expressed content with social distancing measures and online contact	Yes: autistic researchers	Impact on wellbeing
Bozkus-Genc and Sani-Bozkurt (2022)	Qualitative retrospective	Where: Turkey When: September 2020 CHI: 51.49–68.75	Parents: *n* = 8, including 3 of autistic adults: age range: 18–26; 33% female gender; IQ/ID not reported	Comparison group: no; pre-lockdown data: no	Impact COVID-19 (interview; parent-report)	Yes, decrease	Benefits: increased online/phone contact with friends; satisfaction with distance education; disadvantages: weight gain; problems with distance education; opportunities: distance education; vaccine priority; remote counselling service; stretching curfew for autistic adults	Yes: survey design	Impact on wellbeing
Brondino et al. (2021)	Quantitative	Where: Italy When: COVID-19 infection: May 2020 CHI: 85.42–68.75 COVID-19 vaccination side effects: April 2021 CHI: 81.73–80.77	Autistic adults with ID: *n* = 36; median age = 29.5 years; age range: 24–41 years; 22% female sex; Staff daycare centre/farm community: *n* = 35; median age = 38; age range: 28–48 years; 71% female sex	Comparison group: yes; pre-lockdown data: no	COVID-19 infection (blood tests; medical database) and COVID-19 vaccination side effects (UKU side-effect rating scale)	N/A	Autistic adults with ID equally likely infected by COVID-19 as non-autistic staff members; autistic adults less likely to display typical COVID-19 symptoms; most frequent side effect of COVID-19 vaccination in autistic adults was light fever (*n* = 7)	Not reported	Effects COVID-19 virus
Brondino et al. (2020)	Quantitative prospective	Where: Italy When: February 19–March 4, 2020 CHI: 22.02–64.58	Autistic adults with ID: *n* = 18; M age = 22.7; 39% female gender	Comparison group: no; pre-lockdown data: yes	Impact of COVID-19 restrictions on problem behaviours (ABC; daycare worker-report)	No	Stable level of problem behaviours during first 2 weeks of restrictions; preventive measures such as trekking may have counteracted potential negative effects	Not reported	Impact on wellbeing
Bundy et al. (2022)	Quantitative and qualitative; prospective and retrospective	Where: UK When: T0: February–March 2020; T1: May–July 2020 CHI: T0: 14.88–60.71 T1: 61.61–61.90	Autistic adults: Quantitative retro-/prospective study: *n* = 70; M age = 39.8; range: 21–65; 83% female sex; 76% female gender; 30% no employment; 19% living alone; Qualitative study: *n* = 133; M age = 42.9; range: 20–72; 63% female sex; 63% female gender; 30% not employed; 26% living alone	Comparison group: no; pre-lockdown data: yes	Retrospective: Impact of COVID-19 (non-standardized survey; self-report) Prospective: Depression, anxiety, stress (DASS-21; self-report); impact of COVID-19 (qualitative interview, self-report)	Retrospective: yes, decrease; Prospective: yes, increase (less anxiety and stress)	Quantitative study: lower T0 depression, anxiety and stress, access to support, social engagement; set new routine, social activities, less uncertainty about lockdown predicted better outcome; Themes qualitative study: changes in the social world, living with uncertainty, disruptions to self-regulation and barriers to fulfilling basic needs	Yes: autistic researchers	Impact on wellbeing
Cage and McManemy (2022)	Quantitative and qualitative; retrospective	Where: UK When: November–December 2020 CHI: 67.86–76.31	Autistic students: *n* = 70; M age = 24.2; 71% female gender Non-autistic students: *n* = 315; M age = 21.4; 85% female gender	Comparison group: yes; pre-lockdown data: no	Impact of COVID-19 (non-standardized open question; self-report)	Yes, decrease	Similar effects of the pandemic on personal life and study reported by autistic and non-autistic students, including feelings of social isolation, difficulties adjusting to online education and poorer well-being	Yes: survey design	Impact on wellbeing
Critchley et al. (2021)	Qualitative retrospective	Where: UK When: First UK lockdown (March 26–July 4, 2020) CHI: 60.71–66.67	Parent-sibling dyads: *n* = 8 Autistic adults: *n* = 5; age range: 18–33 years; 20% female gender; IQ/ID not reported	Comparison group: no; pre-lockdown data: no	Impact of COVID-19 (interview; parent- and sibling-report)	Unclear	Benefits: closer family ties; feeling safe at home Disadvantages: fear of and lack of understanding COVID-19; limited access to educational and social support	Not reported	Impact on Wellbeing
Davidson et al. (2020)	Quantitative and qualitative; retrospective	Where: UK When: May 22–June 15, 2020 CHI: 58.63–65.77	Autistic adults: *n* = 51; no other information available	Comparison group: no; pre-lockdown data: no	Impact of COVID-19 (non-standardized survey; self-report)	Yes, decrease	72% reported decline in mental health; negative stressors were ‘uncertainty about the future’ and ‘change in routines’; ‘Having to spend more time with household members’, ‘social distancing’ and ‘not being able to go to work/education’ rendered both negative and some positive responses; establishing new routines and making plans were coping strategies	Yes: survey design	Impact on wellbeing
Ferguson et al. (2021)	Quantitative retrospective	Where: US When: May 27–July 10, 2020 CHI: 63.30–65.18	Caregivers: *n* = 339; 93% mothers; Autistic individuals: *n* = 335; M age = 13.4 years; age range: 2–45; 22% female gender	Comparison group: no; pre-lockdown data: no	Types and satisfaction with services (non-standardized survey; caregiver-report)	N/A	Vocational services (*n* = 12; 67% telehealth) and group home/assisted living (*n* = 7; 71% in person) were rated average/neutral; high dissatisfaction with disability services at university (*n* = 3; 100% telehealth)	Not reported	Impact on services
Fridell et al. (2022)	Qualitative retrospective	Where: Sweden When: June–August 2020 CHI: 54.76–51.19	Autistic adults: n = 13; M age = 33.4; age range: 26–55 years; 77% female gender; 31% not employed; 54% living alone	Comparison group: no; pre-lockdown data: no	Impact of COVID-19 (interview; self-report)	Yes, decrease	Many reported a worsening of mental health; some reported reduced access to support, while others continued to receive support; many reported a need for socializing, although some enjoyed the reduced social demands	Not reported	Impact on wellbeing
Friedman (2021)	Quantitative prospective	Where: US When: 2018–2020	Adults with IDD (*n* = 2284) including autistic adults (15.8%)	Comparison group: yes; pre-lockdown data: yes	Quality of life (POM interviews; healthcare database)	N/A	Averaged over 2019 and 2020, autistic adults were more likely to have best possible health and live in integrated settings compared to non-autistic adults	Not reported	Impact on wellbeing
Gibbs et al. (2021)	Quantitative	Where: Australia When: March–October 2020 CHI: 22.02–61.90	Autistic adults: Quantitative study: *n* = 16; M age = 39, age range: 21–76; 63% female gender; Qualitative study: n = 6	Comparison group: no; pre-lockdown data: no	Telehealth experience and satisfaction (survey and interview; self-report)	N/A	81% felt comfortable with telehealth; tele-assessments convenient option; overall positive experience, technology has some limitations	Not reported	Impact on services
Goldfarb et al. (2022)	Quantitative and qualitative; prospective	Where: Israel When: T0: September 2019–January 2020 T1: April–May 2020 T2: October 2020 CHI: T0: 0.00–4.17 T1: 76.79–70.24 T2: 75.60–50.0	Autistic adults: T0: *n* = 34; M age = 29.4, age range: 20–54 years; 12% female gender; 0% not employed; T1: *n* = 23; M age = 30.2; age range: 20–49 years; 17% female gender; 30% not employed; T2: *n* = 10; 20% female gender	Comparison group: no; pre-lockdown data: only employment	Employment status (self-report); Emotional distress (GHQ-12; self-report); work satisfaction (MSQ and basic psychological need satisfaction and frustration (work); self-report)	Yes, decreased mental health in case of unemployment	Quantitative: 30% lost employment at T1 vs T0; employment predicts stable (vs increase in) emotional distress; Qualitative: stable work routines protect mental health; working from home reduces risk of sensory overload; being physically at work may promote social contact and boost mood	Not reported	Impact on wellbeing
Gómez-Ramiro et al. (2021)	Quantitative retrospective	Where: Spain When: December 14 2019–June 12, 2020 (before/after lockdown) CHI: 0.00–52.98	Adults admitted to psychiatric emergency service before lockdown: *n* = 1208 including 4 autistic adults; M age = 40.8; range not reported; 51% female gender Adults admitted to psychiatric emergency service during lockdown: *N* = 750 including 14 autistic adults; M age = 41.8; 44% female gender	Comparison group: yes; pre-lockdown data: yes	Psychiatric emergency admission (medical records)	Yes, decrease	Overall decrease (of 38%) of admissions during lockdown compared to before; significant increase of autistic adults admitted to psychiatric emergency services during lockdown (pre-lockdown: 0.3%; during lockdown: 1.9% of total number of adults admitted)	Not reported	Impact on wellbeing
Halstead et al. (2021)	Quantitative; Retrospective and prospective	Where: UK When: T0: November 2019–January 2020 T1: April 2020 T2: June 2020 CHI: T0: 0.00–14.88 T1: 60.71–61.61 T2: 63.39–67.56	Autistic adults: n = 95; M age = 36.9; age range: 18–65; 62% female gender; 28% not employed	Comparison group: no; pre-lockdown data: yes	Prospective (all time points): sleep quality (PSQI; self-report); sleep arousal (PSAS; self-report); Retrospective: sleep quality (non-standardized survey; self-report)	Retrospective: yes, decrease; Prospective: yes increase	Retrospective: worsening of sleep quality, 65% reported the pandemic impacted sleep with 37% waking up exhausted, 34% not being able to get to sleep and only 5% sleeping better (at T1); majority felt more anxious and depressed at T1 compared to T0, about half felt more anxious and depressed at T2 compared to T0; Prospective: improvement of sleep quality and improved cognitive sleep arousal during lockdown compared to before	Not reported	Impact on wellbeing
Hansford et al. (2022)	Quantitative retrospective	Where: Canada When: March 2020–July 2021 CHI: 7.74–68.45	Adults with IDDs: *n* = 833 (including 78 with autism), M age = 44.4 years; range not reported; 45% female sex	Comparison group: yes; pre-lockdown data: no	Positive COVID-19 test (home care database)	N/A	Autistic adults have equally high chance of positive COVID-19 test as other IDD groups; older adults and those living in congregate settings higher chance of positive COVID-19 test	Not reported	Effects COVID-19 virus
Harris et al. (2021)	Qualitative retrospective	Where: US When: Not reported CHI: -	Autistic adults (self-report): *n* = 7; 43% 21–30 years; 29% female gender; Caregivers: *n* = 12 of 12 autistic adults: 75% 21–30 years; 17% female gender	Comparison group: no; pre-lockdown data: no	Telehealth experience (interview; self-report and caregiver-report)	N/A	Benefits telehealth: more comfortable staying at home; reduced COVID-19 risk; similar/better communication; Disadvantages: technological issues; physical health check not possible	No	Impact on services
Hedley et al. (2021)	Quantitative and qualitative; retrospective	Where: Australia When: June–October 2020 CHI: 54.17–61.90	Autistic adults: Quantitative study: *n* = 103; M age = 41.7; age range: 21–71 years; 57% female gender; 36% not employed; 22% living alone; Qualitative study: *n* = 72; M age = 43.1; range not reported; 64% female gender	Comparison group: no; pre-lockdown data: no	COVID-19 impact (CIS; self-report); wellbeing (PWI-A; self-report); depression (PHQ-8); suicide risk (SBQ-R)	Qualitative: yes, decrease	Quantitative: Higher wellbeing predicts lower suicide risk; COVID-19 impact unrelated to suicide risk; qualitative: 60% reported a moderately/severely negative impact of the pandemic on wellbeing, 22% a mild positive impact, and 18% neutral impact	Not reported	Impact on wellbeing
Heyworth et al. (2022)	Qualitative retrospective	Where: Australia When: May 19–June 29, 2020 CHI: 59.82–47.92	Autistic parents: *n* = 35; M age = 42.7 years; age range: 32–54; 94% female gender; 37% not employed; 0% living alone; Autistic children: *n* = 55; M age = 10.2 years; age range: 4–25; 40% female gender	Comparison group: no; pre-lockdown data: no	Impact of COVID-19 (interview; self-report)	Yes, decrease	Themes: initially COVID-19 was a break from stressful daily life and neurotypical social standards; accumulation of stress by burden of care and COVID-19 stress; decreased mental health and inability to ask for or inaccessibility of (formal) support; closer family ties	Yes: autistic researchers	Impact on wellbeing
Koyama et al. (2022)	Quantitative	Where: US When: March 2020–June 2021 CHI: 13.69–63.72	Hospitalized autistic COVID-19 patients: *n* = 1525; 88% 18–65 + years; 24% female gender Hospitalized COVID-19 patients without IDD: N = 634.161; 99% 18–65 + years; 49% female gender	Comparison group: yes; pre-lockdown data: no	Severe COVID-19 outcomes; 30-day readmission; increased length of stay in hospital (medical database)	N/A	Autistic patients with COVID-19 had a significantly higher risk of ICU admittance and a longer hospital stay (> 40%) than patients without IDDs	Not reported	Effects COVID-19 virus
Krieger et al. (2021)	Quantitative	Where: Israel When: February 11, 2021 (data retrieved) CHI: 71.79	Autistic individuals: *n* = 16,406; M age = 14.4; range not reported; 66% < 16 years; 20% female sex; Sex and age matched non-autistic comparison group: *n* = 16,406	Comparison group: yes	COVID-19 infection and hospitalisation and morbidity due to COVID-19 (insurance database)	N/A	40–60 years: 2.05 times more likely to get COVID-19; > 16 years: 2.20 times more likely to be hospitalized; males higher COVID-19 infection rate and hospitalisation than females	Not reported	Effects COVID-19 virus
Levante et al. (2022)	Quantitative retrospective	Where: Italy When: April–May 2020 CHI: 73.81–68.75	Parents: *n* = 43; M age = 54.8; 81% mothers; Autistic adults: *n* = 43; M age = 24.8; range not reported; 23% female sex; 74% with ID	Comparison group: no; pre-lockdown data: no	Sleep, negative emotional state, aggression at lockdown (non-standardized survey; parent-report); behavioural problems before and at lockdown (non-standardized survey; parent-report)	Yes, decrease	Better sleep–wake routine predicts better outcome; more behavioural problems at lockdown compared to before lockdown	Not reported	Impact on wellbeing
Lois Mosquera et al. (2021)	Qualitative; retrospective	Where: Spain When: T0: Pre-lockdown (before March 16 2020) T1: During first lockdown (March 16–June 21 2020) CHI: T0: N/A T1: 50.30–42.56	Autistic adults: *n* = 5; M age = 30.2 years; age range: 23–37; 60% female gender; 20% living alone; 40% not employed	Comparison group: no; pre-lockdown data: Yes	Navigating social world and wellbeing before and during lockdown (interview; self-report)	Yes, decrease	Lockdown period contained positive daily experiences including reduced social pressure, but also increased societal rejection of autistic adults (who were allowed to go out), feelings of vulnerability and lack of support	Not reported	Impact on wellbeing
Lugo-Marin et al. (2021)	Quantitative; prospective and retrospective	Where: Spain When: T0: pre-lockdown T1: At least 8 weeks after lockdown CHI: -	Autistic adults: *n* = 35; M age = 32.8 years; range not reported; 34% female gender; Parents/partners: *n* = 32; M age = 52.7 years; 69% mothers; 81.2% female gender	Comparison group: yes; pre-lockdown data: yes (psychopathology)	Prospective: psychopathology (SCL-90-R; self-report); Retrospective: stress (non-standardized survey, self-report)	Prospective: yes, increase; Retrospective: yes, increase	Being younger (< 30 years) and autistic predicted better outcome; reduced perceived stress and symptoms of psychopathology in autistic adults during lockdown	Not reported	Impact on wellbeing
Maljaars et al. (2022)	Quantitative and qualitative; retrospective	Where: Belgium (63%), the Netherlands (12%), UK (26%) When: June 20–September 14, 2020 CHI: Belgium: 53.57–55.95; Netherlands: 55.95–50.60; UK: 65.77–61.01	Autistic adults: *N* = 196; M age = 41.5 years; range not reported; 70% female gender; 30% living alone; 28% not employed; non-autistic adults: *n* = 228; M age = 51.5 years; 70% female gender; 7% living alone; 18% not employed	Comparison group: yes; pre-lockdown data: no	Impact COVID-19 (non-standardized survey; self-report); stress (PSS-10; self-report); perceived stress change and coping (qualitative; self-report)	Yes, decrease (self-perceived change in stress)	Quantitative: being non-autistic predicted better outcome; qualitative: engaging in (new) activities, social support, relaxation techniques, (new) routines; exercise, less attention to pandemic new, cognitive coping strategies predict better outcome	Yes: survey design	Impact on wellbeing
Manning et al. (2020)	Quantitative retrospective	Where: US When: March 26–May 4, 2020 CHI: 63.10–66.67	Autistic adults (self-report): *n* = 12; M age = 30.8 years; age range: 20–52; 67% not employed Autistic individuals (caregiver-report): *n* = 459; M age = 11.8 years; age range: 2–46	Comparison group: no; pre-lockdown data: no	Disruption to daily activities and stress (non-standardized survey; self-report and caregiver-report)	Yes, decrease	On a scale from 0 (no disruption/stress) to 10 (severe disruption/stress), 58% of self-reporting adults reported ≥ 7; 74% of caregivers reported that the autistic individual experienced high disruption (≥ 7) and 50% reported high stress (≥ 7); according to caregivers stress level was higher among older autistic individuals	Not reported	Impact on wellbeing
Matthews et al. (2021)	Quantitative retrospective	Where: US When: March 18–September 30, 2020 CHI: 51.79–61.90	Autistic adults: *n* = 2; age range: 19–23; 50% female gender	Comparison group: no; pre-lockdown data: no	Telehealth satisfaction (survey; self-report)	N/A	Both adults felt comfortable and they appreciated a remote diagnostic assessment; one thought the result of an in person assessment would have been different	Not reported	Impact on services
Mupaku et al. (2021)	Qualitative retrospective	Where: South-Africa When: June 2020 CHI: 78.57–76.19	Young adults with ID leaving youth care: *n* = 6 including 2 autistic adults: age range 18–20 years; 50% female gender; 100% with ID; Caregivers: *n* = 3, including 1 mother of 2 autistic adults	Comparison group: no; pre-lockdown data: no	Impact of COVID-19 (interview; self-report and caregiver-report)	Yes, decrease	Participants noticed a regression of independence, increased anxiety and depression, more quality time together	Not reported	Impact on wellbeing
Nistico et al. (2022)	Quantitative retrospective	Where: Italy When: May 4–May 8, 2020 CHI: 73.51–73.51	Autistic adults: *n* = 45; M age = 37.5 years; range not reported; 33% female gender; 9% not employed (before lockdown) Non-autistic gender- and age matched adults: *n* = 45; M age = 40.2 years; 47% female gender; 7% not employed (before lockdown)	Comparison group: yes; pre-lockdown data: no	Depression, anxiety and stress (DASS-21; self-report); trauma-related symptoms (IES-R; self-report); stress (PSS; self-report); impact of lockdown (non-standardized survey; self-report)	Yes, increase (reduced tiredness)	Being non-autistic predicted better outcome on standardized measures (lower depression, anxiety, stress and trauma-related symptoms); autistic adults were less tired during lockdown as compared to before, while non-autistic adult reported similar tiredness; autistic adults more comfortable (better psychological wellbeing) with social distancing measures than non-autistic adults	Not reported	Impact on wellbeing
Nistico et al. (2022)	Quantitative prospective	Where: Italy When: T0: May 4–May 8, 2020 T1: February–March 2021 CHI: T0: 73.51–73.51 T1: 75.77–81.73	Autistic adults: *n* = 45; M age (at T1) = 38.3 years; age range: 20–60; 33% female gender; 22% not employed (at T1)	Comparison group: no; pre-lockdown data: no	Depression, anxiety and stress (DASS-21; self-report); trauma-related symptoms (IES-R; self-report); stress (PSS; self-report); impact of lockdown (non-standardized survey; self-report)	Yes, decrease	From the first lockdown in 2020 to the second lockdown in 2021 autistic adults increased in symptoms of depression, anxiety, stress and trauma	Not reported	Impact on wellbeing
Nollace et al. (2020)	Quantitative	Where: France When: March–April 2020 CHI: 32.44–71.43	Autistic adults with ID: *n* = 16; M age = 20.8 years; age range: 12–43; 24% female sex	Comparison group: no; pre-lockdown data: no	COVID-19 symptoms (medical database)	N/A	11 out of 16 with suspected COVID-19 tested positive for COVID-19; most people displayed common COVID-19 symptoms (e.g. respiratory infection signs, diarrhoea and fatigue)	Not reported	Effects COVID-19 virus
Oakley et al. (2021)	Quantitative retrospective	Where: Europe (15 countries) When: April 7–May 30, 2020	Autistic people: *n* = 346 including 326 adults Caregivers: *n* = 955 including 850 caregivers of autistic adults	Comparison group: no; pre-lockdown data: No	COVID-19 health and social care policy (policy review per country); impact of COVID-19 (survey, self-report/caregiver-report)	N/A	Policy review: most countries did not prioritize COVID-19 testing for autistic individuals despite higher COVID-19 risk; COVID-19 care in neuropsychiatric settings may be suboptimal; triage policy not always adjusted to autism, in some countries cognitive impairments were an exclusion criterion for ICU admittance Survey: half of autistic individuals (55%) and caregivers (51%) reported that reasonable adjustments (e.g. room with less stimuli) to COVID-19 testing were not possible; 42% of autistic individuals and 79% of caregivers reported their usual daily support ceased	Yes: Autistic researchers	Impact on services
Oomen et al. (2021)	Quantitative and qualitative; retrospective	Where: Belgium (50% of sample), the Netherlands (23%) and UK (27%) When: April 3–May 7, 2020 CHI: Belgium: 63.10–67.26; Netherlands: 63.69–63.69; UK: 60.71–61.61	Autistic adults: *n* = 613; M age = 38.4 years; age range: 18–78 years; 68% female sex; 29% living alone; 37% not employed; Comparison: *n* = 431; M age = 38.4; age range = 18–81; 72% female sex; 12% living alone; 11% not employed	Comparison group: yes; pre-lockdown data: no	Depression (adapted PHQ-9; self-report); anxiety (adapted GAD-7; self-report); (pandemic-related) worries (non-standardized survey; self-report)	Quantitative: yes, decrease	Quantitative: being non-autistic predicted better outcome (i.e. reduced increase in depression and anxiety symptoms); themes qualitative study: (continued) professional support, clearer information and rules, (new) routines, social support network, social cohesion, reduced sensory and social overload predict better outcome	Yes: survey design	Impact on wellbeing
Pellicano et al. (2021)	Qualitative retrospective	Where: Australia When: May 19–June 29, 2020 CHI: 59.82–47.92	Autistic adults: *n* = 44; M age = 39.1 years; age range: 23–69; 64% female gender; 41% living alone; 19% not employed Parents: *n* = 84 including 35 autistic parents: M age = 42.7 years; age range: 32–54; 91% female gender; 0% living alone; 37% not employed	Comparison group: no; pre-lockdown data: no	Impact of COVID-19 (interview; self-report)	Yes, decrease	Themes: release of social pressure during the pandemic; loss and missing of social experiences; decline of mental health	Yes: autistic researchers	Impact on wellbeing
Perera et al. (2020)	Quantitative	Where: England (95%) and Ireland (5%) When: June 8–19, 2020 CHI: 66.96–65.77	Adults with ID who died of COVID-19: *n* = 66 including 6 autistic adults; Median age = 64 years; age range: 31–88; 41% female sex	Comparison group: yes; pre-lockdown data: No	COVID-19 deaths (medical database)	N/A	Within a group of adults with ID, autism was not a high-risk factor for dying of COVID-19	Not reported	Effects COVID-19 virus
Pfeiffer et al. (2022)	Quantitative prospective	Where: US When: T0: January 3–February 29, 2020 T1: March 20–April 2, 2020; T2: April 20–May 3, 2020 CHI: T0: 0.00–11.90 T1: 59.52–63.10 T2: 66.67–66.67	Autistic adults: *n* = 6; M age = 23.5 years; age range: 21–27; 33% female sex; 0% living alone; 17% not employed at T0, 33% not employed at T1/2	Comparison group: no; pre-lockdown data: yes	Daily activities and transportation modes (non-standardized survey, self-report); mobility (GPS tracking)	N/A	Number and variety of activities decreased during lockdown; number of trips, transportation modes, and time spent outside the home decreased during lockdown	Not reported	Other
Reynaud et al. (2021)	Quantitative retrospective	Where: France When: During first lockdown (March 17–11 May 2020) CHI: 71.43–68.45	Autistic adults: *n* = 207; M age = 34.5 years; range not reported; 56% female sex; 26% living alone; General population sample: *n* = 1,652; M age = 35.4 years; 77% female sex; 19% living alone	Comparison group: yes; pre-lockdown data: no	Sleep quality and sleep–wake rhythms (non-standardized survey, self-report)	Yes, decrease	Autistic adults reported poorer sleep quality, more irregular sleep–wake patterns, longer sleep duration, less physical activity, less daylight exposure and more screen time; Autistic and comparison adults reported similar decrease in sleep quality and more irregular sleep–wake patterns during lockdown	Not reported	Impact on wellbeing
Riese and Mukherjee (2021)	Quantitative retrospective	Where: UK When: October 2020–February 2021 CHI: 62.20–79.29	Autistic adults: *n* = 120; M age not reported; age range: 26–55; 60% female gender; 33% living alone	Comparison group: no; pre-lockdown data: no	Impact of COVID-19 (non-standardized survey; self-report)	Yes, decrease	Areas most impacted by the pandemic were routines, rigidity, sensory perception, non-verbal communication and theory of mind; about 80% reported that the lockdown had a negative effect on their mental health	Yes: survey design	Impact on wellbeing
Scheeren et al. (2022)	Quantitative prospective	Where: Netherlands When: T0: February 28–March 15, 2020 T1: April 24–May 4, 2020 T2: October 30–November 8, 2020 CHI: T0: 13.10–42.86 T1: 63.69–63.69 T2: 57.74–62.50	Autistic adults: T0–T1–T2: *n* = 299; M age T0 = 45.1 years; range not reported; 57% female gender; T1–T2: *n* = 448; M age T1 = 46.6 years; 56% female gender; 53% no employment at T1; Non-autistic adults T1–T2: n = 70; M age T1 = 49.0 years; 69% female gender; 30% not employed at T1	Comparison group: yes; pre-lockdown data: yes	Stress (PSS-10; self-report); loneliness (DG-LS-6; self-report)	No change	Stress and loneliness of autistic adults remained stable across three timepoints (before lockdown, at first and at second lockdowns); being non-autistic, older, without prior mental health conditions, low COVID-19 concerns and high perceived social support predicted a better outcome	Not reported	Impact on wellbeing
Schott et al. (2022)	Quantitative retrospective	Where: US When: Based on pre-pandemic data (2008–2012)	Autistic adults (without ID): *n* = 31.101; M age = 30.9 years; 28% female sex; Autistic adults with ID: *n* = 52,049; M age = 33.1 years; 29% female sex; Claimants without autism, ID and MHC: *n* = 683,778; M age = 34.5 years; 71% female sex; all groups: 20–64 years	Comparison group: yes; pre-lockdown data: yes	COVID-19 risks (insurance database)	N/A	More factors increasing risk of (more severe) COVID-19 infection in autistic adults (e.g., living in residential facility, comorbid physical conditions such as obesity) compared to non-autistic adults	No	Effects COVID-19 virus
Shea et al. (2022)	Quantitative retrospective	Where: US When: March 29–July 26, 2021 CHI: 60.27–55.01	Autistic adults COVID-19 vaccine acceptant: *n* = 131; M age = 32 years; 23% female gender Autistic adults COVID-19 vaccine hesitant: *n* = 37; M age = 30 years; 27% female gender	Comparison group: no; pre-lockdown data: no	COVID-19 vaccine acceptance (non-standardized survey; self-report)	N/A	78% received or planned to get a COVID-19 vaccine; COVID-19 vaccine acceptance was higher among those living in more populous areas where the Democrats won the presidential elections and those reporting an increase in loneliness during the pandemic; most frequently reported reason for vaccine acceptance: desire to protect others (73%); most often reported reason for vaccine hesitance: concern about vaccine safety (70%)	Not reported	Effects COVID-19 virus
Spain et al. (2021)	Quantitative and qualitative; retrospective	Where: England, UK When: April 28–May 31, 2020 CHI: 61.61–61.01	Professionals in autism care/research: *n* = 37; age and gender not reported	Comparison group: no; pre-lockdown data: no	Impact of COVID-19 (non-standardized survey; professional-report)	Yes, decrease	73% noted a major or complete loss of services and therapy for autistic individuals; difficulties dealing with uncertainty and change were observed; some experienced a relief of social pressure; decrease in mental health; gradual/phased return to normal advised	Yes: survey design	Impact on wellbeing
Spain et al. (2022)	Quantitative and qualitative retrospective	Where: UK (70%), US (10%), other countries (20%) When: August–September 2020 CHI (UK): 65.48–62.20	Professionals in autism diagnostics: *n* = 52; 60% working with adults; age and gender not reported	Comparison group: no; pre-lockdown data: no	Services and telehealth (non-standardized survey; professional-report)	N/A	52% clinicians reported service disruption (temporarily closing); 58% reported longer waiting times for assessment; 85% adapted the standard diagnostic process due to the pandemic, including telehealth; wearing protective material during in-person meetings may affect social interaction and assessment; views about the validity and reliability of remote autism assessments varied, with some finding it adequate and others inappropriate	Yes: survey design and autistic researchers	Impact on services
Taylor et al. (2021)	Quantitative prospective	Where: US When: T0: March 11–20, 2020; T1: May 18–27, 2020 CHI: T0: 25.60–59.52 T1: 66.67–66.67	Young autistic adults employed at T0: *n* = 144; M age = 26.8 years; age range: 18–35; 47% female gender	Comparison group: no; pre-lockdown data: yes	Employment status and perceived impact of employment change (non-standardized survey; self-report); depression (BDI-II; self-report)	Yes, decrease in mental health in case of job loss/reduction	26.4% reported job loss or reduced hours at T1; stable employment (vs job loss/reduction) status predicts stable (vs increase) depressive symptoms over time	Not reported	Impact on wellbeing
Tso et al. (2022)	Quantitative	Where: Hong Kong When: February 2020–November 2021 CHI: 68.45–76.49	Autistic students: *n* = 29; age range: 18–26; 21% female gender Non-autistic students: *n* = 32; age range: 18–26; 19% female gender	Comparison group: no; pre-lockdown data: no	Facial recognition of masked and unmasked faces (experiment, self-report)	N/A	Study 1: When unmasked faces were learned first, masked faces were more difficult to recognize for both autistic and non-autistic adults, no difference in performance; Study 2: when masked faces were learned first, autistic adults recognized unmasked faces worse than non-autistic adults	Not reported	Other
Valderrama et al. (2022)	Quantitative and qualitative retrospective	Where: Canada When: September 2020 CHI: 65.48–63.10	Adults with autism and/or disabilities: *n* = 55 including (at least) 29 autistic adults (53%); age and gender not reported Caregivers of autistic people and/or disabilities: *n* = 279	Comparison group: no; pre-lockdown data: no	Support and services (non-standardized survey; self-report/caregiver-report)	N/A	59% of adults receiving healthcare received telehealth services; 52% of those needing home support did not receive it; large majority received delivery services if needed; about half did not receive COVID-19 information adapted to their needs; 65% of adults perceived their life during the pandemic as quite or extremely stressful; high perceived social support and access to (tele)healthcare and home support predicted less stress	Yes: survey design	Impact on services
Vereijken et al. (2021)	Qualitative retrospective	Where: Netherlands When: March 15–June 15, 2020 CHI: 42.86–55.95	Mothers of adults with IDD: *n* = 7; M age = 60.7 years; Adults with IDD: *n* = 7, including 1 autistic adult; M age = 29.7 years; 100% with ID	Comparison group: no; pre-lockdown data: no	Motives to move child with IDD from residential care to parental home (interview; mother)	N/A	Motives for mothers to take their adult child with IDD home during the start of the pandemic: looking out for their child’s best interest, wanting to be close to them and protecting them. A mother of an autistic adult reported that a protective environment would only make it harder for people such as her daughter to return to society	Not reported	Impact on wellbeing
Weinstein et al. (2021)	Quantitative	Where: Israel When: February 2021: 1.5 months after start mass vaccination plan CHI: 88.45–72.02	Autistic adults: *n* = 5540; M age = 25.5 years; range not reported; 20% female sex; Age and sex matched comparison group: *n* = 5540	Comparison group: yes	COVID-19 vaccination prevalence (healthcare database)	N/A	Autistic individuals more frequently vaccinated (51% vs 29%); biggest vaccination difference in 21–40 age group (62% vs 29%); In 60 + age group no vaccination difference (71% vs 71%)	Not reported	Effects COVID-19 virus
Weir et al. (2022)	Quantitative prospective	Where: UK (54%), US (11%), other countries (35%) When: July 2019–January 2021 CHI (UK): 0.00–81.67	Autistic adults: *n* = 1285; M age = 41.3 years; age range: 16–70 + ; 64% female sex; 60% female gender Non-autistic adults: *N* = 1364; M age = 38.4 years; age range: 16–70 + ; 63% female sex; 52% female gender	Comparison group: yes; pre-lockdown data: yes	Quality of healthcare (non-standardized survey; self-report)	N/A	Autistic adults reported to receive poorer quality healthcare (e.g. healthcare professional did not understand described symptoms or gave insufficient time) than non-autistic adults both before and during the pandemic; experienced quality of healthcare remained stable before/during the pandemic for autistic and non-autistic adults	Yes: survey design	Impact on services
White et al. (2021)	Quantitative retrospective	Where: US When: T0: March 20–April 1, 2020 T1: April 23–29, 2020 CHI: T0: 59.52–63.10 T1: 66.67–66.67	Caregivers whose autistic child received services at T0: *n* = 3,502; M age = 43.4 years; 93% female sex; Autistic individuals: *n* = 3,502 (including 411 adults); M age = 11.8 years; range not reported; 20% female sex	Comparison group: no; pre-lockdown data: no	COVID-19 impact on services (non-standardized survey; caregiver-report)	N/A	Majority reported disruption in services; 59% of autistic adults used remote special education services, 21% remote medical services, 53% remote mental health services; 68% of caregivers of adults reported significant/moderate benefits of telemental health services; 62% reported severe/moderate negative effects of disrupted services on their adult child	Not reported	Impact on services
Wood et al. (2022)	Qualitative retrospective	Where: UK When: July–December 2020 (before/during second lockdown) CHI: 67.56–76.31	Autistic teachers: *n* = 21; Median age = 41 years; age range: 25–56; 90% female sex; 62% female gender; 0% not employed	Comparison group: no; pre-lockdown data: no	Impact of COVID-19 measures on work experience of teachers (interview; self-report)	Individual differences	Themes: Less sensory overload, reduced social obligations, technological challenges and benefits of working remotely, work-home imbalance, ability to manage work changes depend on timing and communication	Yes: autistic researcher and autistic consultant data interpretation	Impact on wellbeing

*ADHD* attention deficit hyperactivity disorder, *BDI-II* Beck Depression Inventory-II, *CIS*, COVID-19 Impact Scale, *DASS* Depression Anxiety Stress Scale, *DG-LS-6* De Jong Gierveld Loneliness Scale-6, *GAD-7* Generalized Anxiety Disorder-7, *GHQ-12* General Health Questionnaire-12, *GPS* global positioning system, *ICU* intensive care unit, *ID* intellectual disability, *IDD* intellectual and developmental disabilities, *IES-R* Impact of Event Scale-Revised, *MSQ* Minnesota Satisfaction Questionnaire, *N*/*A* not applicable, *ODD* oppositional defiant disorder, *PHQ-8* Patient Health Questionnaire-8, *POM* Personal Outcome Measures, *PSAS* Pre-Sleep Arousal Scale, *PSQ-I* Pittsburgh Sleep Quality Index, *PSS* Perceived Stress Scale, *PWI-A* Personal Wellbeing Index-Adult, *SBQ-R* Suicide Behaviour Questionnaire-Revised, *SCL-90-R* Symptom Checklist-90-Revised, *SI* Stringency Index, *UKU* Udvalg for Kliniske Undersøgelser

^a^The containment and health index (range: 0–100) indicates the strictness of COVID-19 government policies at a given time and place, with a higher score indicating a stricter policy (derived from: https://ourworldindata.org/covid-stringency-index). It is based on the following indicators: closures of schools, workplaces, and public transport, cancellation of and restrictions of public events/gatherings, stay-at-home orders, public information campaigns, restrictions on internal movements, international travel controls, COVID-19 testing policy, extent of COVID-19 contact tracing, face coverings and COVID-19 vaccine policy. If a country used different restrictions in different areas, the most stringent policy was used to calculate the containment and health index. In case specific dates are not reported in the research paper, the first and last day of the reported month are used to calculate the containment and health index

^b^The category ‘Not employed’ is a broad category and may include unemployed people seeking work, unemployed people not seeking work (e.g. full time parents), and people with a disability unable to work. If mentioned in the study sample description, students and retired participants were excluded from the ‘not employed’ category

## Results

### Study Selection

**Fig. 1:**
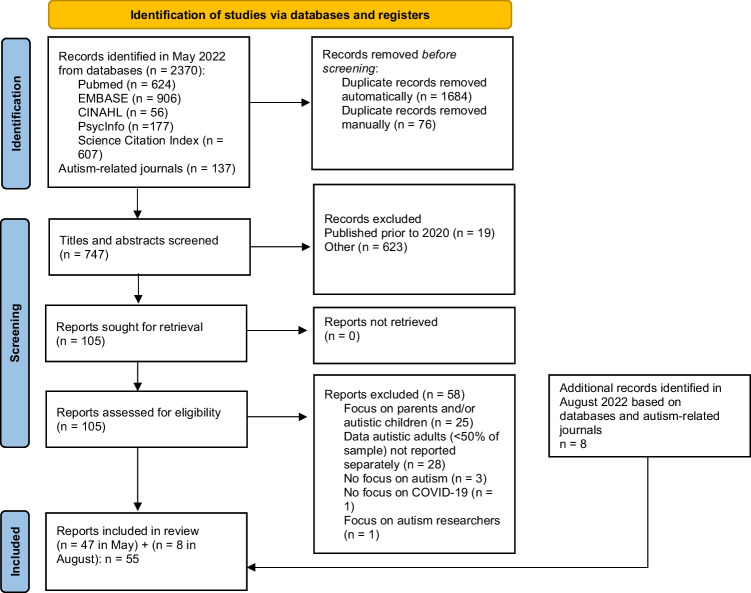
PRISMA flow diagram of the paper selection process. PRISMA flow diagram derived from: Page MJ, McKenzie JE, Bossuyt PM, Boutron I, Hoffmann TC, Mulrow CD, et al. The PRISMA 2020 statement: an updated guideline for reporting systematic reviews. BMJ. 2021;372:n71

During the initial search (May 2022), we identified 747 abstracts. All abstracts were screened for eligibility by AMS. Almost half (*n* = 337; 45%) were independently screened by both AMS and LC, for which there was excellent inter-rater agreement (98%). Disagreements (*n* = 6; 2%) were resolved by discussion. Of the 747 abstracts, we selected 105 (14%) for a full paper read. The same two researchers read all 105 papers to determine final eligibility. Disagreements (*n* = 6 papers; 6%) were resolved through discussion, leading to a final selection of 47 (45%) papers. During the second search (August 2022), we identified an additional eight eligible papers, yielding a final list of 55 studies (see Fig. 1 [15, 16]).

### Critical Appraisal

We assessed the quality of eligible studies using the Mixed Methods Appraisal Tool (MMAT, [17]). Each study was assessed in terms of five methodological quality criteria, which differed according to study methodology (quantitative, qualitative and mixed-methods). For each item, the study was rated ‘yes meets criteria’, ‘no does not meet criteria’ or ‘can’t tell’, where relevant information was not reported. EP rated all eligible studies (*n* = 55), half of which (*n* = 27; 49%) were independently coded by MH, with excellent agreement (88%) (LC rated papers for which EP and MH were also authors). Disagreements were discussed and resolved by consensus. The MMAT discourages allocating a numerical score, preferring a narrative description of a paper’s quality (see Supplementary Table 1). No studies were excluded based on their MMAT assessment, but study quality is applied in the interpretation of the findings.

### Study Themes and Types

We categorised the 55 eligible papers as follows: 1) direct effects of the COVID-19 virus on Autistic adults including infection risk, hospitalisation, death and vaccination rates (*n* = 10); 2) effects of COVID-19 restrictive measures on care, support and services for Autistic adults (*n* = 11); 3) COVID-19-related effects on the wellbeing of Autistic adults (*n* = 32); and 4) other (*n* = 2) (see Table 1). Of the 55 studies, 30 (55%) were quantitative, 13 qualitative (24%) and 12 reported mixed-methods (22%). Thirty-five studies (64%) were, at least partly, based on Autistic adults’ self-reports, 10 (18%) on proxy-reports (caregivers or clinicians) and the remaining 10 (18%) on secondary analyses of medical/insurance databases. Eighteen studies (33%) included a non-autistic comparison group. Thirteen studies (24%) included data collected prior to pandemic onset. Most studies (*n* = 34; 62%) did not report on Autistic community involvement in the research process, and of those studies that did, most reported modest (*n* = 11; e.g. review of the survey) or no (*n* = 2) involvement of community members (see Table 1). In eight studies (15%), it was reported that Autistic researchers contributed to the research.

### Demographic Data

Most included studies took place predominantly in the USA (*n* = 13; 24%) and the UK (*n* = 12; 22%), followed by Italy (*n* = 5; 9%) and Australia (*n* = 4; 7%). Thirty-one studies (56%) collected data in (multiple) European countries (including the 17 UK and Italian studies). Three studies took place in Israel, one in Canada, one in South-Africa, one in Turkey and one in Hong Kong (China). No studies presented data on Autistic adults in Central or South-America. Most (*n* = 34; 62%) collected data at least partly between March and May 2020 (i.e. during the earliest phase of the COVID-19 pandemic and before COVID-19 vaccines were available). The following policy rules applied in most jurisdictions during this time, although their implementation and enforcement varied substantially [7]: physical distancing; wearing face masks; disinfecting hands; cancellation of public events; restriction of international travel; closure of schools, non-essential shops, and restaurants and staying at, and working from, home. Seventeen studies (32%) collected data during a later phase, and three studies did not report timing of data collection. One study, examining risk for COVID-19 infection [13•], based their conclusions solely on pre-pandemic data.

Only nine studies (16%) focussed, at least in part, on Autistic adults with intellectual disability [13•, 18, 19, 20, 21, 22, 23, 24, 25]. For the 35 studies (64%) including Autistic adults’ self-report data, it is likely that participating adults did not have a co-occurring intellectual disability, since self-report requires the ability to understand and respond to questions (orally or in writing), usually without support. The male-to-female gender/sex ratio varied greatly across studies, with the lowest proportion of women (17%) in an employment study [26] and the highest proportion (94%) in a study with Autistic parents [27]. In studies including self-reports (*n* = 35), there was a preponderance of Autistic females (either female gender and/or sex) with 19 (54%) sampling > 50% Autistic females. In the 20 studies without any direct participation by Autistic adults (e.g. through the use of proxy-reports or medical records), the highest proportion of Autistic females was 39% [22] (excluding one study, where the single Autistic participant was female [25]). In 12 of 55 studies (22%), Autistic adults’ gender/sex was not reported, mostly because either professionals were the primary informant [28, 29, 30] or the sex ratio was described only for a larger group of participants with an intellectual or developmental disability (IDD) [18, 31].

### What Has Been the Impact of the COVID-19 Pandemic on Autistic Adults?

#### Direct Effects of the COVID-19 Virus

A large-scale study in Israel (*n* = 32,812; 44% ≥ 16 years) reported that Autistic adults were two times more likely to be infected by COVID-19, compared to sex- and age-matched adults and also two times more likely to develop serious illness, resulting in higher hospitalisation rates [32]. Similarly, Autistic adult COVID-19 patients in the USA — just like those with an IDD — were shown to have a higher risk of Intensive Care Unit admittance and a significantly longer hospital stay, compared to their baseline group of COVID-19 patients without an IDD [33]. The causes for higher infection rates and more severe outcomes among Autistic versus non-autistic adults are not definitive but appear to be linked to contextual factors (e.g. living in a residential facility, receiving services in the home from outside caregivers) [13•, 18].

A review of COVID-19 policies in multiple European countries [34] demonstrated that, despite concerns about an increased risk, most countries did not prioritize COVID-19 testing for Autistic people. Furthermore, survey data showed that 55% of Autistic adults and 51% of caregivers of Autistic adults reported that reasonable adjustments to COVID-19 testing (e.g. a support person or sensory accommodations) were not made possible [34]. Moreover, it appears that Autistic people with an intellectual disability may have been discriminated against, as some countries used cognitive impairments as an exclusion criterion for Intensive Care Unit admittance in case of triage [34].

Given Autistic adults’ increased risk of infection, some advocates called for prioritization of COVID-19 vaccination in this group. During the early phase of a mass vaccination campaign in Israel, Autistic adults were reported as being more likely to be vaccinated, compared to a sex- and age-matched comparison group, particularly in the 21–40 age group (Autistic: 62%; non-autistic: 29%; [35]). In a US-based study, most Autistic adults (78%) were said to have had received or planned to get a COVID-19 vaccine [36].

In sum, Autistic adults appear to be at increased risk for COVID-19 infection and show poorer outcomes following infection compared to non-autistic adults. Despite this risk, COVID-19 testing has not been prioritized for this group. Given that only two studies in this review reported about COVID-19 vaccinations for Autistic adults, it remains uncertain whether COVID-19 vaccinations were consistently prioritized.

#### Impact on Services and Supports

Autistic adults, caregivers and professionals reported major disruption of services for themselves or those they supported during the COVID-19 pandemic [29, 34, 37]. The barriers to accessing healthcare, which were already excessive for many Autistic people prior to the pandemic [38, 39, 40], were deemed even greater during the pandemic [41•]. Due to physical distancing and stay-at-home orders, many services shifted to remote delivery to ensure continuity of care. Experiences of so-called ‘telehealth’ services varied substantially, linked to the nature of the service. Telehealth services were reported to be an acceptable alternative to in-person service delivery for primary care [42] and diagnostic assessments [43], largely because virtual appointments can minimise barriers to care for Autistic adults (e.g. less stress negotiating travel and waiting rooms; [39]). Yet, remote mental health services were not accepted or preferred by everyone [44••, 45, 46] and were advised against in cases of high psychological distress or intellectual disability [41•]. Autistic adults who accessed remote mental health services reported challenges with sensory issues [44••, 46], body awareness [44••, 46], technological issues [41•, 42, 43, 46] and effective communication with their therapist [44••, 46]. Some reported experiencing discontinuity of care as a result [46], and those who felt they did *not* benefit from telehealth services early in the pandemic were more likely to experience mental health distress 2 months later, compared to those who felt they benefited [47•]. These mixed findings suggest that, even though telehealth services may be an acceptable and welcome alternative for some Autistic adults, the specific nature of service delivery should depend on individual preference.

#### Impact on Wellbeing

Of the 32 studies examining the pandemic’s effects on wellbeing, 13 (41%) were quantitative, 10 (31%) qualitative and nine (28%) used a combination of qualitative and quantitative methodologies. Most studies assessed the pandemic’s effects on the wellbeing of Autistic adults (either via self-report or proxy-report) in a retrospective way, that is, by asking participants to reflect on and estimate the impact of the pandemic, compared to their memory of pre-pandemic experience. Almost all retrospective studies indicated an overall decrease in wellbeing during the pandemic. Only 10 (31%) studies were prospective, where the wellbeing of Autistic adults was assessed repeatedly over time. These prospective studies revealed mixed experiences, demonstrating either overall *stability* of psychopathology symptoms, loneliness and stress in Autistic adults during the first months of the pandemic [47•, 48, 49], an overall *decrease* in psychopathology symptoms [44••] or an *increase* in acute psychiatric distress, as evidenced by increased psychiatric emergency admissions of Autistic adults before and during lockdown [50].

Perhaps unsurprisingly, there were large individual differences reported within these prospective studies, even for those reporting negligible overall effects — suggesting that, for some Autistic adults, the COVID-19 pandemic and its restrictions may have had positive effects on their wellbeing, while for others, it had negative effects. This variability was confirmed by studies adopting in-depth qualitative retrospective approaches, which also revealed potential reasons for this variability. Most studies reported differences *between individuals*, with some Autistic adults highlighting benefits of the restrictions (e.g. relief of pressures from the external world, control over sensory stimulation, ability to spend quality time with family) and others reporting serious and damaging effects of these same restrictions (e.g. loss of social contacts and friendships, no access to professional support, and loss of independence) [44••, 45, 46, 51, 52••]. Unsurprisingly, some of these studies also reported substantial differences *within individuals*, with some Autistic adults experiencing both positive *and* negative effects of the stay-at-home orders at different points during the pandemic [27, 44••, 46, 52••, 53]. This latter finding might provide one explanation for the apparent stability in psychological wellbeing reported by several prospective, quantitative studies [47•, 48, 49]. Another possibility is that such stability is driven more by the consistently deeply unsatisfactory quality of life and poor mental health experienced by many Autistic adults pre-pandemic (see [14], for review) than the impact of the pandemic itself.

### Which Individual and/or Contextual Factors During the COVID-19 Pandemic Are Associated with a More Positive Outcome for Autistic Adults?

#### Individual Factors

Many studies identified individual characteristics that were reportedly predictive of better mental health in Autistic adults during the pandemic, including being male gender/sex [47•, 49], older [47•, 48], having relatively good mental health prior to the pandemic [44••, 47•, 48, 49], low COVID-19-related stress [27, 48, 49], high perceived social support [46, 48, 52••] and being able to maintain routines or develop new ones [44••, 52••, 54]. Caution is warranted, however, as these effects (particularly regarding gender/sex and age) were not consistently replicated [44••, 48].

#### Contextual Factors

There were several contextual factors that appear to have had a positive effect on Autistic people’s mental health or wellbeing during the pandemic, including (continued) access to (professional) support [27, 44••, 52••, 53], reduced pressure to conform to societal rules [27, 46, 51, 53], reduced sensory overload or greater control over the sensory environment [44••, 52••, 55], absence of COVID-19 infection [47•], strong social connections and activities (including maintaining contact with friends, family and community online) [44••, 46] and clear and accessible public health messaging with regard to COVID-19-related information and rules [52••]. Societal inclusion and acceptance are also likely protective factors of Autistic adults’ mental health in general [53].

## Discussion

Through our scoping review, we have demonstrated that Autistic adults are likely to have been disproportionately impacted by the COVID-19 pandemic in several ways: [1] they were at increased risk of contracting COVID-19 and developing serious illness; [2] they often struggled to access critical services and supports as a result of stay-at-home restrictions implemented in many jurisdictions; and [3] despite some positive uplift due to reduced external pressures, many experienced challenges to their mental health — particularly those who were female, had pre-existing mental health issues and had limited social connections and support. These findings are consistent with the pandemic’s detrimental effects on disabled people more broadly, referred to as the “triple jeopardy” [1].

While these findings are already deeply concerning, our review likely significantly *underestimates* the negative impact of COVID-19 on Autistic adults’ health and wellbeing, since most reviewed studies relied on selective samples. For instance, almost all studies were conducted in high-income countries, meaning we know virtually nothing of the experiences of Autistic adults residing in low- and middle-income countries, many of whom may be undiagnosed and with limited or no access to appropriate mental health care [56, 57]. We also know little of the experiences of Autistic adults from seldom-heard groups in autism research, including those who face economic hardship, are of minority racial/ethnic backgrounds, use non-traditional forms of communication, and/or have an intellectual disability. Autistic adults identifying with one or more of these groups were vastly under-represented in the studies we identified, which makes it difficult to draw conclusions about their experiences of COVID-19. It is likely, however, that those who are multiply marginalized and those living in low- and middle-income countries will have responded far worse than the identified studies suggest.

Aside from the sampling issues outlined above, many of the identified studies were also of relatively poor methodological quality. While there was an urgent need to understand the impact of COVID-19 on the Autistic and autism communities, many studies relied, often for logistical and practical reasons, on retrospective designs as well as measures of often questionable reliability, validity and generalisability. For instance, several studies relied on bespoke rating scales, single items or failed to report a measure’s reliability within an Autistic sample (see also Supplementary Table 1). Many studies also did not account for potential confounding factors, especially the extent and nature of COVID-19-related restrictions at the time of study. Despite clear research recommendations formulated early on in the pandemic [58], concerns regarding the scientific rigor of the COVID-19 literature are not limited to autism research [59, 60]. Such concerns are worrying nevertheless because conclusions based on less-than-sound science can pose significant challenges to clinical and policy-relevant decision making and may also reduce trust in autism science.

Our review may also be limited in other ways. We acknowledge that our search strategies may not have been exhaustive, especially regarding the exclusion of non-English language studies, and that our review included several studies of our own, which may have increased the risk of bias. It also may have over-represented issues faced during the first phase of the pandemic (likely due to lags between study completion and publication), rendering it important to examine later and long-term impacts of COVID-19 and its associated restrictions. Further limitations of the reviewed studies likely transcend the pandemic situation. For example, level of community involvement was rarely reported or was very modest (e.g. questionnaire review), indicating many missed opportunities to actively learn from and be guided by the needs and expertise of the Autistic community [58]. Such opportunities may have been particularly pertinent during the unprecedented pandemic situation.

### Lessons Learned

Despite the aforementioned concerns, there are several important lessons we can draw from this review on the future care and support of Autistic adults. First, Autistic adults have been disproportionately affected by the COVID-19 pandemic — both by the effects of the virus itself and the social measures used by governments to curtail it. We therefore need a distinct strategy to support Autistic adults during times of crisis and beyond. Such a strategy must be deeply informed by, and actively co-produced with, the Autistic community to ensure it is responsive, respectful and relevant to Autistic adults’ lives.

Second, continued access to effective, knowledgeable and tailored care and services was vital for Autistic adults’ wellbeing during the pandemic. We need greater investment in the development — and rigorous testing — of services and supports that are tailored to the needs, preferences and abilities of individual Autistic adults, particularly those that optimise the person-environment fit [61]. Autistic adults should also be actively involved in making decisions about specific, formal supports (in terms of personnel and delivery) and be able to shape the systems and policies enabling access to these supports, even during health crises.

Third, although our review revealed much variability in the extent and nature of the impact of the pandemic on Autistic adults’ mental health, many experienced acute loneliness and social isolation, with some reporting severe mental health distress. This finding challenges prevailing stereotypes of autism — that is, that Autistic people prefer a life of self-isolation. In future, more concerted efforts, ideally led by Autistic-led organisations, must be made to implement and maintain social support structures for the Autistic community [46, 48], especially during times when social supports are otherwise curtailed.

Finally, few studies in our review reported actively including Autistic adults or their allies in the research process. Participatory autism research is still in its infancy, with researchers often reporting that it can be time-consuming and challenging [62]. The pandemic may well have exacerbated these challenges [63]. Researchers should seek to deepen their involvement with the Autistic community so that they can respond rapidly and flexibly in time of crises such as this one. Doing so will ensure that future research, clinical services and care are aligned with the needs and priorities of Autistic people [27], accelerate translational research and enable Autistic people to have their perspectives and experiences fully taken into account.

## Supplementary Information

Below is the link to the electronic supplementary material.

Supplementary file1 (DOCX 21 KB)

## Data Availability

Data are available upon reasonable request.

## Footnotes

1 In the autistic community, identity-first language e.g. ‘autistic person’, is often preferred to, and considered less stigmatizing than, person-first language e.g. ‘person with autism’ (2–3; although see 4). We therefore use identity-first language throughout this paper. We also use ‘Autistic’ with a capital A. Similar to the Deaf community, many autistic adults spell Autistic with a capital A to emphasise their belonging to a shared community and to recognise their struggles to achieve acceptance and social justice.
