# Supplementary material for: Impact of the COVID-19 Pandemic on Autistic Adults: a Scoping Review
Source: Curr Dev Disord Rep. 2023 Jan 31;10(1):92–122. doi: 10.1007/s40474-023-00268-6 (PMC9887236; doi:10.1007/s40474-023-00268-6)
Supplement: Supplementary file 1 — Supplementary file1 (DOCX 21 KB) [file 40474_2023_268_MOESM1_ESM.docx]

**Supplementary Table 1.**

*Results of the study quality assessment using the Mixed Methods Appraisal Tool (MMAT).*

| **Study type** | **Quality criteria** | **N studies meeting criteria** | **Reasons studies did not meet criteria (i.e., received a “no” or “can’t tell” rating)^a^** |
| --- | --- | --- | --- |
| **1. Qualitative (n=25)** | 1.1 Is the qualitative approach appropriate to answer the research question? | N=22 (88%) | Research questions were focused on the quantitative portion of the data and did not specify how qualitative methods would be used to address the research question (n=3) |
|  | 1.2 Are the qualitative data collection methods adequate to address the research question? | N=16 (64%) | Relied on a single open-ended question (n=5) within an online survey to capture people's experiences; or used several open-text questions but did not provide sufficient detail about the extent of data available for analysis (n=4) |
|  | 1.3 Are the findings adequately derived from the data? | N=12 (48%) | Insufficient detail provided on the nature of the analytic process (n=7); no inter-rater reliability reported for studies using content analysis (n=2); lack of sophistication to analysis (n=4); or no formal analysis of qualitative data (n=1) |
|  | 1.4 Is the interpretation of results sufficiently substantiated by data? | N=13 (52%) | Number of quotes used to substantiate the identified themes was inadequate (n=10); or no clear themes identified (n=2) |
|  | 1.5 Is there coherence between qualitative data sources, collection, analysis and interpretation? | N=18 (72%) | Relationship between the research questions, qualitative methods and study findings was unclear and sometimes overly descriptive in nature (n=7) |
| **3. Quantitative non-randomised (n=27)** | 3.1 Are the participants representative of the target population? | N=3 (11%) | Target population was autistic people, however, methods (only survey/interview) were a barrier to participation for those with intellectual or language difficulties (n=14); target population was autistic people, however, those with intellectual disability were over-represented (n=2); sample was predominantly female (n=5); sample was predominantly employed (n=2); insufficient information reported on participant characteristics to determine representativeness of sample (n=7) |
|  | 3.2 Are measurements appropriate regarding both the outcome and intervention (or exposure)? | N=19 (70%) | Used bespoke questionnaire with single items of dependent variable of interest (n=2); relied on retrospective rating scales to examine change in dependent variable of interest (n=4); no estimates of reliability reported for sample on standardised measures (n=5); reported reliability estimates for standardised scales but results showed poor reliability (n=1); insufficient information provided to judge appropriateness of measures (n=1) |
|  | 3.3 Are there complete outcome data? | N=11 (41%) | Amount of missing data was either not reported or was unclear (n=13); >20% of data missing on a dependent variable of interest (n=3) |
|  | 3.4 Are the confounders accounted for in the design and analysis? | N=16 (59%) | Accounted for no or few potential confounders in the design and/or analysis (n=11) |
|  | 3.5 During the study period, is the intervention administered (or exposure controlled) as intended? | N=16 (59%) | Did not provide sufficient contextual information related to ‘exposure’ of COVID-19 itself, or of COVID-19-related restrictions (n=9); did not provide sufficient information on degree of adherence to COVID-19-related restrictions in sample population (n=6) |
| **4. Quantitative descriptive (n=15)** | 4.1 Is the sampling strategy relevant to address the research question? | N=14 (93%) | The sampling strategy went beyond the target population (n=1) |
|  | 4.2 Is the sample representative of the target population? | N=1 (7%) | Target population was autistic people, however, methods (only survey/interview) were a barrier to participation for those with intellectual or language difficulties (n=3); target population was autistic people, however, those with intellectual disability were over-represented (n=2); data from larger dataset and differences in sampling characteristics between those who responded and those who did not (n=1); insufficient information reported on participant characteristics to determine representativeness of sample (n=6); insufficient information about the sampling characteristics of the target population (n=2) |
|  | 4.3 Are the measurements appropriate? | N=4 (27%) | Used bespoke questionnaire, often with single items of dependent variable of interest (n=5); relied on retrospective rating scales to examine change in dependent variable of interest (n=1); no estimates of reliability reported for sample on standardised measures (n=4); used rating scales/measures that were difficult to interpret (n=2); insufficient information provided to judge appropriateness of measures (n=2) |
|  | 4.4 Is the risk of nonresponse bias low? | N=4 (27%) | Amount of missing data was either not reported or was unclear (n=9); >20% of data missing on a dependent variable of interest (n=2) |
|  | 4.5 Is the statistical analysis appropriate to answer the research question? | N=10 (67%) | Statistical analyses used were inappropriate (n=3); accounted for no or few potential confounders in the design and analysis (n=1); statistical analyses were not directly tied to the research question (n=1) |
| **5. Mixed methods (n=12)** | 5.1 Is there an adequate rationale for using a mixed methods design to address the research question? | N=4 (33%) | The rationale for the use of both qualitative and quantitative methods was not adequately explained (n=8) |
|  | 5.2 Are the different components of the study effectively integrated to answer the research question? | N=3 (25%) | No attempt, or no clear attempt, to integrate the qualitative and quantitative components of the study either in the results or the discussion sections of the paper (n=9) |
|  | 5.3 Are the outputs of the integration of qualitative and quantitative components adequately interpreted? | N=7 (58%) | Inadequate integration of the qualitative and quantitative components during the interpretation of the findings (n=5) |
|  | 5.4 Are divergences and inconsistencies between quantitative and qualitative results adequately addressed? | N=6 (50%) | Divergencies and inconsistencies between the qualitative and quantitative results were not adequately discussed (n=6) |
|  | 5.5 Do the different components of the study adhere to the quality criteria of each tradition of the methods involved?^b^ | N=2 (17%) | The overall quality of one (n=3) or both (n=7) components was low (n=10) |

Notes: ^a^Some studies failed to meet the criterion for more than one of the stated reasons; ^b^The MMAT guidance (Hong et al., 2018) specifies that, for criterion 5.5, the quality of both components should be high for the mixed methods study to be considered to be good quality. If one component is rated high quality and the other rated low quality, the overall rating for this criterion will be of low quality.
